# Clinical Observation of Employment of Umbilical Cord Derived Mesenchymal Stem Cell for Juvenile Idiopathic Arthritis Therapy

**DOI:** 10.1155/2016/9165267

**Published:** 2015-12-06

**Authors:** Liming Wang, Yu Zhang, Hongtao Li, Jingxin Hong, Xiaobo Chen, Ming Li, Wen Bai, Jiangang Wang, Yongjun Liu, Mingyuan Wu

**Affiliations:** ^1^Cell Therapy Center, 323 Hospital of People's Liberation Army, Xi'an 710054, China; ^2^Zhongyuan Union Cell & Gene Engineering Corporation Company, Tianjin 300304, China; ^3^Alliancells Institute of Stem Cells and Translational Regenerative Medicine & Alliancells Bioscience Co., Ltd., Tianjin 300381, China; ^4^Harold Hamm Oklahoma Diabetes Center and Section of Endocrinology and Diabetes in the Department of Internal Medicine, University of Oklahoma Health Sciences Center, Oklahoma City, OK 73104, USA

## Abstract

Juvenile idiopathic arthritis (JIA), known as Juvenile rheumatoid arthritis, is the most common type of arthritis in children aged under 17. It may cause sequelae due to lack of effective treatment. The goal of this study is to explore the therapeutic effect of umbilical cord mesenchymal stem cells (UC-MSCs) for JIA. Ten JIA patients were treated with UC-MSCs and received second infusion three months later. Some key values such as 28-joint disease activity score (DAS28), TNF-*α*, IL-6, and regulatory T cells (Tregs) were evaluated. Data were collected at 3 months and 6 months after first treatment. DAS28 score of 10 patients was between 2.6 and 3.2 at three months after infusion. WBC, ESR, and CRP were significantly decreased while Tregs were remarkably increased and IL-6 and TNF-*α* were declined. Similar changes of above values were found after 6 months. At the same time, the amount of NSAIDS and steroid usage in patients was reduced. However, no significant changes were found comparing the data from 3 and 6 months. These results suggest that UC-MSCs can reduce inflammatory cytokines, improve immune network effects, adjust immune tolerance, and effectively alleviate the symptoms and they might provide a safe and novel approach for JIA treatment.

## 1. Introduction

Juvenile idiopathic arthritis (JIA) is a common rheumatic disease and is the primary cause of disability and blindness in childhood [1]. The main feature of JIA is chronic arthritis accompanied by systemic multiorgan involvement. This disease can be divided into three types: systemic, polyarticular, and oligoarticular [2]. JIA commonly occurs in 2–16-year-old children with characteristics of long-term fever, rash, joint pain, and leukocytosis. It can affect growth and development of the victims and is a formidable disease to treat [3]. New treatment is urgently needed. Mesenchymal stem cells (MSCs) could be harvested from a variety of tissues such as bone marrow, adipose tissue, umbilical cord, placenta, and muscle [4, 5]. MSCs showed the ability to differentiate towards multiple cell lineages including osteoblasts and chondrocytes [6, 7]. On the other hand, MSCs were immunosuppressive and immunoprivileged, display high migration and motility, and could secret cytokines to improve the repair of damaged tissues; therefore MSCs have been used to treat various diseases in clinic trials [8, 9]. Previously we have reported data harvested from adult patients with Active Rheumatoid Arthritis treated by UC-MSC [10]. Here we report the first attempt to our knowledge to use umbilical cord mesenchymal stem cell (UC-MSC) to treat JIA. In this study, 10 cases of JIA patients aged 2–15 were treated with UC-MSC at two time points.

## 2. Materials and Methods

### 2.1. Patients

Ten JIA inpatients were selected from our department dating from October 2011 to November 2012, according to the American College of Rheumatology criteria, ARA [11]. The patients consisted of 6 males and 4 females, aged 2–15 years. The course of disease ranged from 1 to 12 years. Six cases were systemic type JIA, three cases were polyarticular type, and one case was oligoarticular type. The detailed information is listed in Table 1. The patients were evaluated by 28-joint disease activity score (DAS28). All patients were above 3.2. DSA28 were between 3.2 and 5.1 points in eight cases and were above 5.1 points in two cases. Erythrocyte sedimentation rate (ESR) of eight patients was between 40 and 100 mm/h and was above 100 mm/h in the other two cases. C-reactive protein (CRP) was between 50 and 100 mg/L in five cases and above 100 mg/L in the other five. The white blood cell count (WBC) of all the patients was between 10.0 × 10^9^/L and 26.0 × 10^9^/L. All the patients had been treated repeatedly with steroids, nonsteroidal anti-inflammatory drugs (NSAIDS), disease modifying antirheumatic drugs (DMAIDS), and biological agents, but the treatments showed no significantly beneficial effects.

### 2.2. Pretesting

Level of cytokines in peripheral blood was tested before and after treatments. Peripheral blood was collected in procoagulant tube and centrifuged in 3500 rpm/min for 5 minutes. Serum was then transferred into EP tubes. Each tube contained 200 microliters of serum and was saved in −80°C freezer for further testing. BD Multitest_IMK kit was used to detect the level of TNF-*α* and IL-6 in serum, and multifunction streaming LUMINEX 200 was used for analysis and detection.

Regulatory T cells (Tregs) were stained with anti-CD4 (BD, number 340133) fluorescein isothiocyanate, anti-CD25-Allophycocyanin (BD, number 340939), and anti-Foxp3-PE (eBioscience, number 12-4776-42). Isotype control antibodies were used alternatively. Samples were incubated in the dark for 15 minutes and analyzed with flow cytometer (BD, FACS Calibur, USA). Image and data were acquired and saved.

### 2.3. Preparation of the UC-MSCs

UC-MSCs were obtained from Alliancells Institute of Stem Cells and Translational Regenerative Medicine. UC-MSC was harvested according to our previous study [12]. Briefly, the newborn umbilical cord was collected and cut to about 1 mm × 1 mm × 1 mm in dimension and then was digested with 0.1% collagenase and 0.125% trypsin in 37°C for 30 min. Undigested tissues were removed with filters. After filtration, the cells were seeded by 1 × 10^6^/cm^2^ in plastic culture flasks containing DMEM-LG/F12 (Sigma, USA), 5% FCS (Gibco BRL, USA) medium, and then were placed into the incubator. The medium was changed after 5 days and nonadherent cells were discarded. Half medium was changed every 3 days. The cells were able to adhere to the plastic and showed fibroblast-like morphology. Harvested cells were characterized according to our previous study [12], which is suggested by the ISCT [13]. The characterization procedure was performed according to our previous procedure. Cell surface markers such as CD29, CD31, CD34, CD73, CD90, and HLA-DR were analyzed by flow cytometry (BD, FACS Calibur, USA). Isotype-matched normal mouse IgGs were used as controls. Image and data were acquired and saved.

### 2.4. Cell Quality Control of MSCs

All used MSCs will be firstly analyzed for tumor formation with agar cloning assay* in vitro*. HeLa cells were used as positive control. Before injection, MSCs will be investigated with several tests for cell viability, bacteria, fungus, mycoplasma, and endoxin. Cell viability assay was performed by 2% trypan blue staining; the percentage of living cells must be more than 95%. For bacteria detection, bacterial automatic reporting system BacT/ALERT-3D (Merieux) was used. When the detection result is negative (−) and other detection indexes are qualified, the cell culture process will be continued; when result is positive (+), the cells will be abandoned. For fungal detection, acridine orange staining was used with MSC numbers 1–3 × 10^6^. Fluorescence was tested with the 500 nm excitation light. For mycoplasma detection, Hoechst 33258 staining was used and pictures were taken in 340 mm ultraviolet light. For endotoxin detection, samples from all culture media will be detected with Limulus amebocyte lysate gel; the standard is not more than 50 EU/agent. MSCs will proceed to clinical application only if all above test results are qualified. Finally, MSCs with a passage number less than 6 were used for all patients.

### 2.5. Treatment

**Figure 1:**
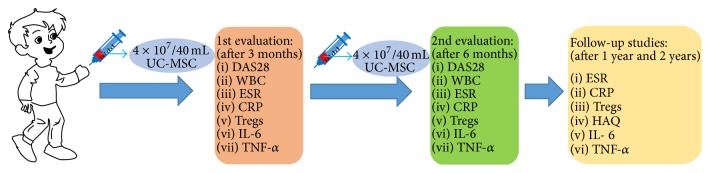
UC-MSC treatment and evaluation procedure. All the 10 patients received infusion with 4 × 10^7^/40 mL MSCs. After 3 months, DAS28 value was measured and peripheral blood of each patient was obtained for WBC, ESR, CRP, Tregs, IL-6, and TNF-*α* measurement. The patients were given the second cell infusion later, and the second measurement was done three months later. Follow-up studies were carried out for 1 year and 2 years.

All the 10 patients received the treatment of UC-MSC with intravenous infusion and the number of cells delivered was 4 × 10^7^/40 mL. At the same time of infusion, the patients were given 2–5 mg (by weight) of dexamethasone for antiallergy. Before the infusion, the patients individually took NSAIDS, DMAIDS, and prednisone in the dose of 10–20 mg per day following the ACR guidelines. All patients were given the second cell infusion three months later. The detailed procedures were shown in Figure 1.

### 2.6. Efficacy Evaluation

DAS28 was used for evaluation of clinical efficacy. A score of DAS28 below 2.6 indicates that disease is in remission, a score between 2.6 and 3.2 points indicates that disease is in low activity, a score between 3.2 and 5.1 points indicates that disease is in moderate activity, and a score above 5.1 points indicates that disease is in high activity. CRP, ESR, inflammatory related factors (TNF-*α* and IL-6), and Treg were evaluated for laboratory efficacy. Follow-up was carried out before and after three months or six months after the cell treatment.

### 2.7. Safety Assessment

Side effect events were recorded during each follow-up. Liver and kidney function, ECG, X-ray, blood pressure, and other indicators were checked regularly in order to detect the side effects and observe the outcome of the situation. Discomfort reaction was graded as the following: 0 is no discomfort, 1 is mild discomfort with no effects on their daily life, 2 is moderate discomfort with negative effects on daily life and learning, 3 is moderate discomfort with daily life significantly affected and immobilized, and 4 is severe discomfort with life-threatening events.

### 2.8. Statistical Analysis

Statistical analysis was performed with Microsoft Office program Excel and SPSS (17.0) software. Measurements were repeated at least three times for each donor. The probability (*P*) value was calculated using *t*-test to assess differences between two groups. Levels of significance were labeled as follows: ^*∗*^ *P* < 0.05 and ^*∗∗*^ *P* < 0.01. Significance was given with the appropriate number of asterisks or in numbers.

### 2.9. Ethic Statement

The use of human umbilical cord materials for isolation, differentiation, and characterization of human umbilical cord derived mesenchymal stem cells was approved by the Ethics Committee in Alliancells Institute of Stem Cells and Translational Regenerative Medicine. The cell therapy was approved by the Ethic Committee in 323 Hospital of People's Liberation Army, and the parents of the patients signed consent to the treatment of UC-MSC.

## 3. Results

### 3.1. Characterization of UC-MSCs

**Figure 2:**
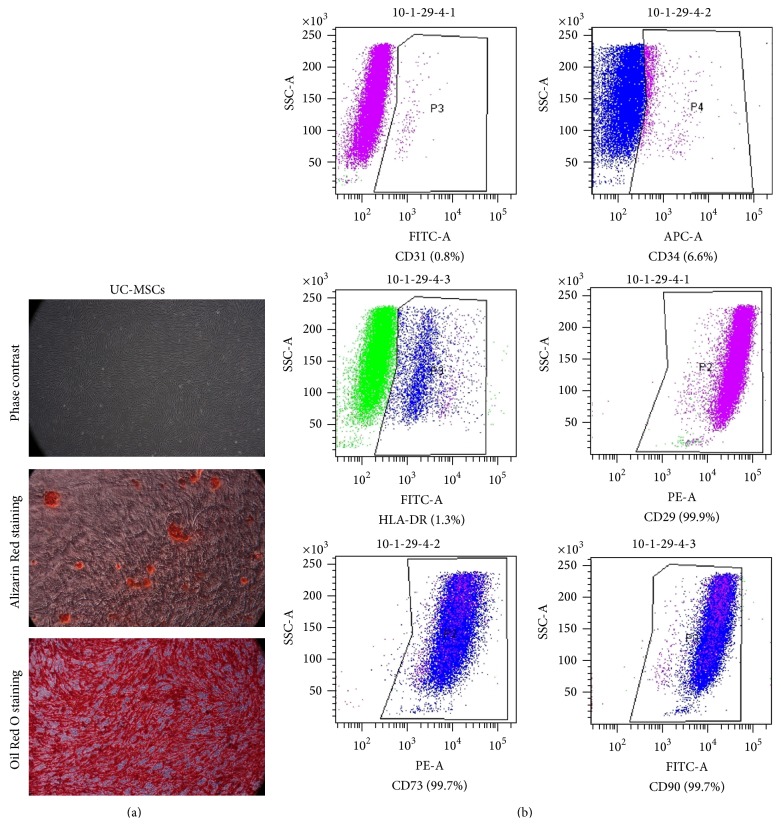
Verification with MSC characters. MSCs were isolated from human umbilical cord materials. Isolated cells displayed fibroblast-like morphology on the plastic surface and were positive for Alizarin Red staining and Oil Red O staining (a). At the same time, isolated cells were analyzed with MSC specific markers by FACS and cells were positive for CD29, CD73, and CD90 but negative for CD31, CD34, and HLA-DR (b).

The isolated UC-MSCs were evaluated if they meet the minimal criteria suggested by the International Society for Cellular Therapy [13], which are plastic adherence, the multipotent differentiation potential into their respective mesenchymal lineages (osteo- and adipogenic), and the expression of the mesenchymal cell markers. To do so, isolated cells showed a fibroblast-like cell morphology and were further differentiated towards adipocytes and osteoblasts as our previous study [12]. The osteogenic differentiation was identified using Alizarin Red S staining of calcium deposits and adipogenic differentiation was verified using Oil Red O staining (Figure 2(a)). In addition to this, FACS analysis was performed for MSC specific markers. Data showed that the isolated cells expressed the mesenchymal cell markers CD29 (99.9%), CD73 (99.7%), and CD90 (99.7%) and as expected, these cells were negative for CD31 (0.8%), CD34 (6.6%), and HLA-DR (1.3%) (Figure 2(b)).

### 3.2. Cell Quality Control of the MSCs

**Figure 3:**
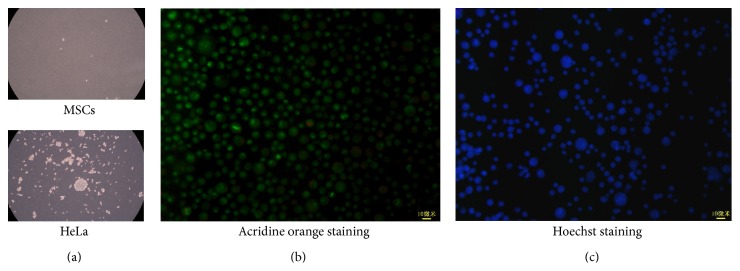
Cell quality of MSCs. MSCs were analyzed with agar cloning assay, acridine orange staining, and hoechst staining, respectively. In the cloning assay, MSCs showed no significant clones compared with HeLa (a). Acridine orange staining showed that only MSCs were stained indicating no fungal contamination (b). Hoechst 33258 staining showed that only MSCs were stained suggesting no mycoplasma contamination (c). This result was taken from one of the ten samples for patients.

The viability of all MSC samples was above 98%. MSCs cultured on the agar showed no clone formation compared with HeLa, which indicates MSCs has very low ability to from tumors* in vitro* (as shown in Figure 3(a)). With acridine orange and hoechst staining, only MSCs were stained with green and blue, respectively, (as shown in Figures 3(b) and 3(c)). Other signals were not detected suggesting cells were not contaminated by fungal and mycoplasma.

### 3.3. DAS Value at 3 Months after the First Treatment

**Figure 4:**
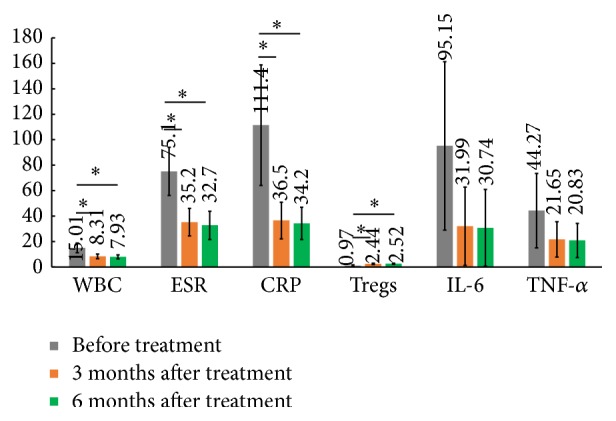
Laboratory measurement before/after UC-MSC treatment in JIA patients. To test the effect of MSC treatment on JIA patients, two-time measurements were done at three months after the first MSC infusion (orange column) and at three months after the second infusion (green column). Values such as WBC, ESR, CRP, Tregs, IL-6, and TNF-*α* were analyzed and differences were compared with the values obtained before the MSC infusion (grey column). WBC, ESR, and CRP showed significant decreases, while Tregs showed a significant increase. The expression of IL-6 and TNF-*α* was declined at three months after the first and second UC-MSC infusion.

No side effects were detected in 10 patients after the UC-MSC treatment. Data for urine routine tests, liver function, and renal function test showed no significant difference. After the first UC-MSC treatment, symptoms such as fever or rash were not observed in all patients, while joint pain was alleviated significantly. Four of the six systemic type patients, two of the three multijoint type patients, and the oligoarticular type patient achieved remission. The DAS28 value of 10 patients was between 2.6 and 3.2. WBC of all the patients declined to 4.0–11.0 × 10^9^/L. In seven patients, it dropped significantly and showed no difference with the normal children. ESR and CRP also declined significantly. ESR value was between 20 and 50 mm/h and CRP value was between 20 and 60 mg/L. On average, Tregs showed significant increase in 10 patients. In eight of the 10 patients, Treg increased very significantly (*P* < 0.01), while in the other two cases it did not increase by comparing pre- and posttreatment. Levels of IL-6 and TNF-*α* decreased with the overall comparison before and after treatment (as shown in Figure 4 with orange column).

### 3.4. DAS Value at 6 Months after the First Treatment

All the patients received the second infusion with UC-MSC at three months after the first infusion. Follow-up observations were carried out three months after the second infusion. DAS28 value was between 2.6 and 3.2. WBC, ESR, and CRP showed a continued declining trend and were significant compared with the values before infusion. However, it is not significant compared to the three months after the first treatment. Tregs continued to increase, but there was no statistical significance compared to the three months after the first treatment. Levels of IL-6 and TNF-*α* decreased compared to the three months after the first treatment and there was no statistical significance (as shown in Figure 4 with green column). The doses of rheumatism drugs, such as NSAIDS and prednisone, were reduced gradually after the second cell treatment.

### 3.5. Follow-Up Study Data on DIA

The 1-year follow-up studies were done for all the patients after the second treatment; four of them were further tracked with 2 years. ESR and CRP value kept in the low level after 1 year. Tregs percentage in the whole T cell family showed no significant changes, HAQ and DAS28 values declined, joint symptoms improved significantly, and the expression of IL-6 and TNF-*α* kept in low levels.

Four patients were kept tracking for 2 years; HAQ and DAS28 values declined. Two of them were unable to walk but now can walk to school independently. Ten patients showed enhanced speed in growth and development with an average of 5–10 cm increase in height. Two patients with necrosis in the femoral head show no aggregations.

## 4. Discussion

JIA is one of connective tissue malignancies, which affects approximately 1 in 1000 children in any given year and with about 1 in 10000 having a more severe form [14]. JIA has been reported to be related to environment, infection, immunology, metabolism, endocrine, and other factors; however the detailed etiology is still unclear. There are two reactions which may coexist for pathogenesis. One is humoral immune response caused by immune complex formed mainly by the rheumatoid factor, which is the major factor causing damage to the articular synovia. The other is cellular immune response, which secretes and releases lymphocyte factors including a variety of globulins, forms immune complexes with rheumatoid factors, and further activates the inflammatory response of the complement system [15]. The traditional treatments are not effective because they cannot completely adjust the immune responses in JIA patients. It may explain why clinical symptoms of JIA occur repeatedly. It also gives our new perspective to look for exploring approaches to repair the patient immune responses. MSCs belong to adult stem cells and mainly derived from early mesoderm and ectoderm. MSC was first found in bone marrow and later in adipose tissue and umbilical cord [16]. MSCs were shown as a potential cell source for regenerative medicine because they are multipotent and more importantly can secret cytokines to regulate immune response and repair the damaged tissue [17, 18]. For example, MSCs were shown to inhibit leukemia/lymphoma cell proliferation* in vitro* and in allogeneic bone marrow transplant in mice [19]. More bodies of evidence were demonstrated to uncover the detailed regulation of MSCs on immune system. The autologous or allogeneic MSCs can significantly inhibit the proliferation and activation of T lymphocytes [20]. UC-MSCs are the undifferentiated primitive cells. UC-MSCs have low immunogenicity because they do not express the mature antigens of the cells and will not be recognized by the immune system [10]. On the other hand, UC-MSCs have a long survival time in the host and can express a variety of cytokines and growth factors such as stem cell growth factor, keratinocyte growth factor, Ghrelin, interleukin-15, and growth hormone to improve the tissue repair [21, 22]. They can also reduce the activation of macrophages and the expression of inflammatory cytokines [23]. Therefore, UC-MSCs are a promising cell candidate for the transplantation treatment of JIA patients [24, 25].

In this study, 10 JIA patients were treated with UC-MSCs. No side effect was observed after MSC infusion. Patients continued to take NSAIDS, DMAIDS, and prednisone. Follow-up data at 3 months after the first treatment showed significant improvement. DAS28 scored lower and reached a low activity of the disease. Laboratory results also showed improvements: WBC, ESR, CRP, and inflammatory cytokines IL-6 and TNF-*α* reduced, whereas Tregs increased significantly. At three months after the second MSC infusion, DAS28 score maintained a low activity level and the laboratory results remained stable. WBC, ESR, CRP, and inflammatory cytokines IL-6 and TNF-*α* decreased while Tregs increased continuously. UC-MSCs can increase the output of mature T cells in the thymus and the number of Tregs in peripheral blood [26]. However, there was no statistical significance between the data from 3 months after the first infusion and 3 months after the second infusion. The reason required further investigations. The dose of antirheumatic drugs decreased with good tolerance. The data for the 1- and 2-year follow-up study showed that the patients with stable condition promote physical development substantially, which indicated the long-term efficacy with UC-MSC treatment.

## 5. Conclusion

Our data demonstrated for the first time that UC-MSC treatment could alleviate the symptom and pain in JIA patients. This might be regulated via repairing patient's immune system by MSC released cytokines. The further studies could be focusing on which factors are the major regulators and downstream transduction pathways.

## Figures and Tables

**Table 1:** List of patient information.

Number	Sex	Age	Course of disease	Symptom	Exterior sign
1	Female	4	2 years	Repeated pain in the joints, mainly in the knee, difficulty in walking	Knee joint deformity, swelling

2	Male	15	5 years	Repeated fever with pain in metacarpophalangeal joint	The right joint deformity

3	Male	14	3 years	Repeated fever with pain in arm and leg joints	

4	Male	9	4 years	Repeated fever with pain in hip and metacarpophalangeal joints	Bilateral femoral head necrosis in phase 2

5	Male	11	5 years	Fever in the early stage, later pain in the joints	Limited squat with knee, restricted movement in elbow

6	Female	10	3 years	Fever in the early stage, later pain in the joints	

7	Female	14	3 years	Pain in the joints	Limited squat with knee, deformity in metacarpophalangeal joints

8	Female	2	1 year	Fever in the early stage, later pain in the joints	The knee joint deformity

9	Male	15	12 years	Repeated fever with pain in arm and leg joints	Metacarpophalangeal joint deformity, necrosis in the right femoral head in phase 2

10	Male	9	4 years	Fever in the early stage, later pain in the joints	Developmental retardation, limited squat with knee
